# Cutting-Edge Research Trends in Colorectal Disease

**DOI:** 10.3390/jcm11041036

**Published:** 2022-02-16

**Authors:** Jacopo Crippa, Donato F. Altomare, Antonino Spinelli

**Affiliations:** 1 IRCCS Humanitas Research Hospital, Via Manzoni 56, Rozzano, 20089 Milan, Italy; jacopo.crippa@humanitas.it; 2 Department of Emergency and Organ Transplantation, University Aldo Moro of Bari, Umberto I Square, 70121 Bari, Italy; donatofrancesco.altomare@uniba.it; 3 Department of Biomedical Sciences, Humanitas University, Via Rita Levi Montalcini 4, Pieve Emanuele, 20090 Milan, Italy

The scientific effort in improving colorectal disease treatment and outcomes has allowed for a continuous shift of burdens that were previously thought to be unassailable. Advancements in the diagnosis and treatment of colorectal illness is encouraged by the constantly increasing incidence of colorectal cancer, diverticular disease and IBD in recent years, especially in Western countries.

One clear example of this progress is represented by the advancement in the perioperative treatment for patient affected by rectal cancer. In the 1990s, radiotherapy was introduced as an adjuvant treatment for patients who received rectal resection for cancer. This revolutionary therapy aimed to reduce local cancer recurrence, which has been a historical threat after rectal surgery. It took over 10 years to prove that radiotherapy could be even more effective if administered as a neoadjuvant treatment. Despite clear evidence, which comprised a manuscript in the *New England Journal of Medicine*, the adoption of such treatment has been slow [1]. One of the various advantages was achieving a complete pathologic response on final pathology, which assessed an originally stage III rectal cancer survival to be comparable to a stage I. Nowadays, Total Neoadjuvant Therapy and Watch and Wait strategies receive constantly growing acceptance by oncologists and surgeons.

Another milestone of the 2000s has been the diffusion of minimally invasive techniques, which surprisingly are still adopted by a minority of surgeons for their demanding training and skills required. Along with perioperative protocols, they radically changed the whole perioperative course of colorectal patients, paving the way for day-surgery operations such as one-day colectomy. Scarless surgery has become an option where extended laparotomy was thought to be the only solution, with unprecedent outcomes on post-operative pain, movement, aesthetic and development of incisional hernia.

Colorectal cancer screening programs have been a key factor for the reduction in colorectal cancer incidence, which has seen a peak in the 1980s and a steady decline, hastened up by the advent of operative colonoscopy in more recent years. Even in the field of early diagnosis, there has been a strong effort to improve the reliability of the screening test and the patient’s compliance by exploring new non-invasive and very promising technologies, including breath biopsy and liquid biopsy looking for specific patterns of volatile organic compounds in the exhaled breath and circulating cancer cells or mutated DNA fragments, respectively [2].

A continuous upgrade of colorectal disease diagnosis and treatment is mandatory if we want to improve patients’ outcomes, and this is not only true when treating a patient with malignant disease but also benign disease. When facing an inflammatory bowel disease (IBD) diagnosis, we can still provide little assurance to patients’ questions about their future [3]. How will this affect my life? Is it proper to delay surgery or should I consider it as a first approach? Will I end up with a definitive ostomy?

Even for a less-feared disease such as diverticular disease, largely affecting the older population [4], the medical and surgical communities still struggle to provide a proper timing or indication to a conservative or operative treatment.

Despite the great incidence of colorectal diseases, this field still presents quite a number of black holes when it comes to scientific evidence on treatment and outcomes. One reason for this is the paucity of high-quality trials and structured research program that has been affecting surgical research for decades. This urge for knowledge has widened as new fields of research have opened. Microbiomes and artificial intelligence are largely undisclosed territories that will pave the way to potential new treatments and may radically change our approach to colorectal diseases and others [5,6]. The interest in micro-organisms grows constantly for their impact on disease and disease prevention, including cancer. Machine learning applied to high-definition imaging has and will continue to revolutionize the way we diagnose patients and address them to the proper treatment. Pixel-magnified microscope slides allow for precise diagnoses and even prognosis. This comes from the artificial intelligence analysis of big data, which are becoming easily available and perfectly fit the broad spectrum of illness of colorectal diseases. Diagnostic and operative techniques during endoscopic colonoscopy allow for early detection and treatment of colorectal neoplasm, sparing the patient from unnecessary invasive treatment. New opportunities are on the way, and it is crucial to keep updated with what will come next.

This Special Issue in the *Journal of Clinical Medicine (JCM)* has the scope to collect the newest advances in the field of colon and rectum disease to present the reader with cutting-edge evidence.

Manuscripts providing readers with upcoming technologies or latest updates on established practices are welcome to enrich this Special Issue.
